# A spurious *fln-2* mutation in a wide variety of commonly used *C. elegans* strains

**DOI:** 10.17912/micropub.biology.001684

**Published:** 2025-07-09

**Authors:** Marina Kniazeva, Gary Ruvkun

**Affiliations:** 1 Molecular Biology , Massachusetts General Hospital, Boston, Massachusetts,; 2 Genetics, Harvard Medical School, Boston, Massachusetts; 3 Molecular Biology, Massachusetts General Hospital, Boston, Massachusetts, United States; 4 Genetics, Harvard Medical School

## Abstract

We describe a *fln-2* mutant allele present in many commonly used *Caenorhabditis elegans* strains. It is present in the *dpy-5 ( e907 )* strain, ancestral to thousands of transgenic strains generated by the *C. elegans* Expression Project. This finding broadens the number of strains affected by the *fln-2* mutation, now impacting thousands of transgenic lines used in diverse studies. This expands on the previous identification of *the fln-2* mutation in a wild-type male stock strain used for outcrossing in genetic studies.

**Figure 1. Background mutation shortens lifespan in Ppgp-5::GFP reporter strain:**
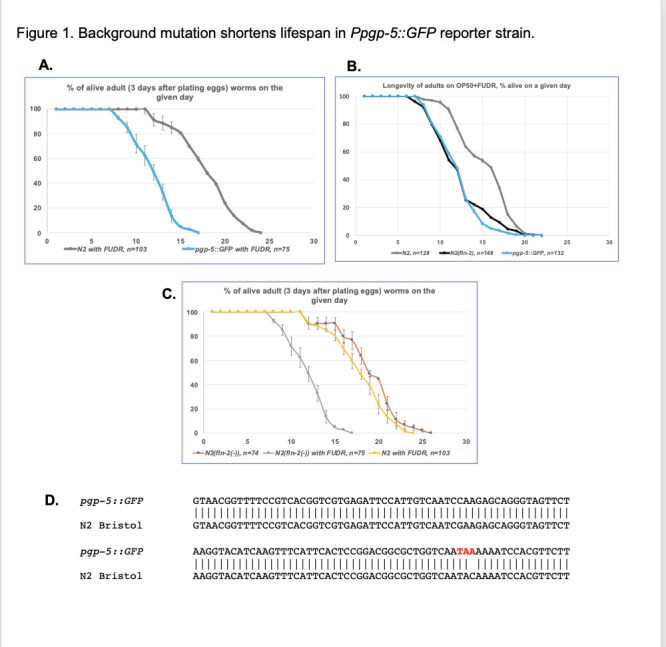
**1A.** Animals carrying *pgp-5::GFP* have reduced life-span due to bursting in the experiments with FUDR-derived inhibition of proliferation. **1B.** The life-span curve is similar in *pgp-5::GFP* strain and “ N2 male” strain carrying *fln-2* Y800* mutation. **1C** . The *fln-2* mutant without FUDR treatment live as long as wild type N2 treated with FUDR. **1D.** DNA alignment of wild type *fln-2* in N2 strain and fln-2 (Y800*) in a strain constructed from a *pgp-5::GFP* reporter gene strain. The aligned DNA sequence refers to the fln-2 locus (and specifically the Y800Stop mutation).

## Description

We investigated *C. elegans* ZIP-2 transcription factor activation using a *pgp-5::GFP* reporter strain. During lifespan assays using FUDR (5-fluoro-2-deoxyuridine), a common method to prevent reproduction and simplify *C. elegans* aging studies, we observed a reduced lifespan and a prominent bursting (rupture) phenotype in this strain ( Fig. 1A ).

Filamins, like FLN-2 , are crucial for organizing the actin cytoskeleton, a network essential for cellular structure, movement, shape changes, and intracellular transport. The *fln-2 ( ot611* ) allele is a C-to-A mutation which causes a premature stop codon instead of the wild type tyrosine 800 (Y800*). This mutation was discovered in a “ N2 male stock” strain used for outcrossing mutants generated through mutagenesis. The strain was distributed from CGC, Caenorhabditis Genetic Center. Notably, in our experiments the bursting and lifespan kinetics in *pgp-5::GFP* strain closely mirrored those of *fln-2 ( ot611 )* mutants (Fig 1B). The onset of rupture events occurred on day 8, with a daily rupture rate of 10-20% of the total population for the subsequent seven days, before dropping to approximately 1% for the remainder of the observation period in both strains. Our data show that *fln-2* mutant without FUDR treatment live as long as wt N2 treated with FUDR ( Fig. 1C )

Zhao et al. (Zhao et al *.* , 2019) reported a *fln-2* Y800* mutation in variety of strains in their collection, finding it in approximately 50% of 50 tested strains, including those from the *C. elegans* Gene Knockout Project (gk and ok alleles, VC and RB prefix strains: 6/10 and 6/6) and the *C. elegans* Expression Project (s alleles: 9/9) (Consortium, 2012; Hunt-Newbury et al *.* , 2007).

Sequencing of our *pgp-5::GFP* strain revealed the identical C-to-A mutation, mutating a TAC tyrosine codon to TAA stop ( Fig. 1D ).

Pedigree analysis of the *pgp-5::GFP* strain revealed its origin: a 300 bp *pgp-5* promoter fragment driving GFP expression, along with a rescuing *dpy-5 (+)* gene, was injected into *dpy-5 ( e907 ) I* mutants, creating the BC10030 strain ( *C. elegans* Expression Project). Subsequent integration of the extrachromosomal array and outcrossing generated the WE5172 strain (McKAY et al *.* , 2003).

The *dpy-5 ( e907 )* strain was originally chosen as a host for easy selection of array-carrying worms, as the co-injected *dpy-5 (+)* construct rescues the *dpy-5* Dpy mutant phenotype to Non-Dpy. *dpy-5 ( e907 )* itself was generated by ^32^ P irradiation of N2 worms in the 1970s at the MRC Laboratory under the supervision of Sydney Brenner. Our sequencing of both BC10030 and WE5172 confirmed the presence of the Y800* mutation. Sequencing two other *dpy-5 ( e907 )* -derived strains, BC14247 and BC10066 (unrelated to *pgp-5::GFP* ), also revealed the *fln-2* mutation.

We conclude that the Y800* mutation was already present in the N2 wild-type stock used to generate *dpy-5 ( e907 )* , probably in the “ N2 male stock" (Zhao et al *.* , 2019).

Given that *dpy-5 ( e907 )* served as the host strain for the creation of approximately 2,000 other *C. elegans* GFP expressing reporters, the potential for widespread dissemination of this mutation is significant.

Our combined data strongly indicate a prevalence of the *fln-2* mutation in strains circulating within the *C. elegans* research community. This finding highlights the importance of thorough strain characterization and careful interpretation of phenotypes, especially those related to cytoskeletal function, secretion, and lifespan.

## Methods

**Longevity assay** :

Gravid worms were bleached, and eggs were rinsed three times with M9 buffer. Eggs were then plated onto assay plates and incubated at 20°C for three days until worms reached adulthood.

Daily, the number of dead worms was counted. Lifespan was calculated in Excel as the number of live worms on each day, normalized to the total number of worms on each plate.

The experiment was performed in triplicate for each strain. Average lifespan and standard error of the mean were plotted on the graphs.

## Reagents

Nematode Growth Media (NGM containing bacto-peptone) (Brenner, 1974) agar plates with streptomycin.

2′-Deoxy-5-fluorouridine (FUDR, F0503, Sigma) stock 40 mM

PCR primers:

fln-2F: GGTGTTCGATTCTGGTCTGG

fln-2R: ACATCGACGAGAAGACAACAC

Sequencing primer:

fln-2SeqPCR: TGTACCCAGAAATTGACAAGATAC

Assay plate preparation for longevity assay: 170 µl of FUDR was mixed in 3 ml of OP50-1 overnight culture and 200 µl were spotted on NGM agar plates (60 mm in diameter). Final FUDR concentration in agar was approximately 30 µM. Plates were let to dry at room temperature overnight.

Worm strains were provided by the Caenorhabditis Genetics Center, CGC:

OP50-1 *E. coli* , streptomycin resistant CGC

N2 Wild type, *C. elegans* CGC

WE5172 *ajIs1 [rCesC05A9.1::GFP + dpy-5 (+)] X* CGC

N2 male stock fln-2 (Y800*) CGC

BC10030 *sEx864 [rCesC05A9.1::GFP + pCeh361]* CGC

BC14247 *sEx14247 [rCes F42G4.3a::GFP + pCeh361]* CGC

BC10066 *sEx900 [rCesC15H9.6::GFP + pCeh361]* CGC
